# Prediction of neddylation sites from protein sequences and sequence-derived properties

**DOI:** 10.1186/1471-2105-16-S18-S9

**Published:** 2015-12-09

**Authors:** Ahmet Sinan Yavuz, Namık Berk Sözer, Osman Uğur Sezerman

**Affiliations:** 1 Biological Sciences and Bioengineering Program, Faculty of Engineering and Natural Sciences, Sabancı University, Tuzla, Istanbul, 34956, Turkey; 2 Department of Genetics and Bioengineering, Faculty of Engineering and Architecture, Yeditepe University, Ataşehir, Istanbul, 34755, Turkey; 3 Department of Biostatistics and Medical Informatics, Faculty of Medicine, Acıbadem University, Ataşehir, Istanbul, 34752, Turkey

**Keywords:** Neddylation, NEDD8, machine learning, support vector machines, post-translational modifications

## Abstract

**Background:**

Neddylation is a reversible post-translational modification that plays a vital role in maintaining cellular machinery. It is shown to affect localization, binding partners and structure of target proteins. Disruption of protein neddylation was observed in various diseases such as Alzheimer's and cancer. Therefore, understanding the neddylation mechanism and determining neddylation targets possibly bears a huge importance in further understanding the cellular processes. This study is the first attempt to predict neddylated sites from protein sequences by using several sequence and sequence-based structural features.

**Results:**

We have developed a neddylation site prediction method using a support vector machine based on various sequence properties, position-specific scoring matrices, and disorder. Using 21 amino acid long lysine-centred windows, our model was able to predict neddylation sites successfully, with an average 5-fold stratified cross validation performance of 0.91, 0.91, 0.75, 0.44, 0.95 for accuracy, specificity, sensitivity, Matthew's correlation coefficient and area under curve, respectively. Independent test set results validated the robustness of reported new method. Additionally, we observed that neddylation sites are commonly flexible and there is a significant positively charged amino acid presence in neddylation sites.

**Conclusions:**

In this study, a neddylation site prediction method was developed for the first time in literature. Common characteristics of neddylation sites and their discriminative properties were explored for further *in silico *studies on neddylation. Lastly, up-to-date neddylation dataset was provided for researchers working on post-translational modifications in the accompanying supplementary material of this article.

## Background

Post-translational modifications (PTMs) are crucial mechanisms in cellular regulation. Many proteins can be modified with small chemical groups or proteins in order to alter their interactions or function for a particular cellular outcome. Studying post-translational modifications provide details for intricacies of protein function and response to various external or internal stimuli.

Neddylation is a highly dynamic and reversible post-translational modification, in which NEDD8 protein is covalently attached to a target lysine residue.

NEDD8, which is one of the ten neural precursor cell-expressed developmentally down regulated (NEDD) genes, encodes the ubiquitin-like modifier of the same name in humans. NEDD8 protein shows ~60% sequence identity with ubiquitin, and it is the most similar protein to ubiquitin among other ubiquitin-like modifiers (Ubls) [1]. In addition to sequence identity, NEDD8 conjugation pathway is also similar to that of the ubiquitin. Resembling SUMO and other Ubl proteins, NEDD8 is synthesized in an immature form and activated by the cleavage of extra amino acids located beyond Gly76 [2]. Gly76 is then able to form a bond with target site's lysine residue [2]. After maturation, NEDD8 follows Ubl protein conjugation pathway with its own specific enzymes (Additional File 1 Figure S1). NEDD8 proteins then can be removed by NEDD8 isopeptidases, making neddylation a dynamic and reversible process [2].

Neddylation may affect lifespan, function and structure of target protein profoundly by altering the 3D surface, stimulating conformational change, and recruiting NEDD8-binding proteins [2]. Additionally, neddylation may also cause further changes in subcellular localization.

Neddylation target proteins are observed to be located predominantly in the nucleus [3]. The most characterized targets of neddylation belong to cullin protein family, whose members act as scaffold proteins of multisubunit E3 ubiquitin ligases called cullin-RING ligases [4]. Therefore, neddylation mainly acts as a regulator of ubiquitin-protein ligases [3]. Secondly, neddylation affects cell-cycle regulation, transcriptional regulation and signal transduction indirectly [5]. Therefore, aberrations in the neddylation pathway or target sites have been observed in many complex diseases, such as cancer, Alzheimer's, and Parkinson's [6-8]. Besides, severe disruptions in the neddylation pathway are shown to be lethal in many organisms [1].

While neddylation performs significant roles in cellular processes, recognition of target sites and specificity of neddylation is still unclear. Identification of neddylation target sites experimentally is an expensive and labour-intensive process. However, there are no previously reported neddylation site motifs or published neddylation site prediction tools available. Therefore, there is an obvious need for *in silico *study of neddylation and prediction of neddylation target sites.

In this work, we have developed a novel method to identify neddylation target sites for the first time using sequence properties, evolutionary conservation, hydrophobicity, disorder and other physicochemical properties of neddylation sites. Additionally, we have provided a list of experimentally verified neddylation sites in supplementary information accompanying this article, which can be used as a benchmark set in further neddylation site prediction studies.

## Results

**Figure 1:**
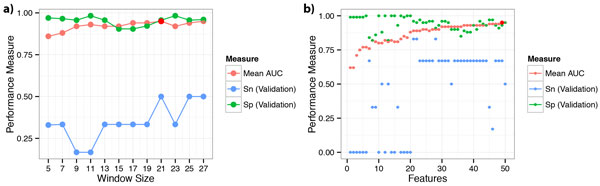
**Optimisation of window size and feature selection**. (a) Effect of window size on mean classification AUC. Mean AUC of 100 repeats of 5-fold stratified cross validation was reported. Two standard errors are shown as error bars. (b) Feature selection using mRMR and incremental feature selection strategy. Number of features to be retained was determined using the mean classification AUC of 100 runs of 5-fold stratified cross validation as the main performance measure. X-axis represents the number of features used in classification. Two standard errors are represented by error bars in the graph. Maximum AUC was found to be 0.95 at 49 features.

Initially, we have identified the optimal sequence window length using maximum feature count of 50. Fine-tuned SVM classification results indicated that the highest average AUC was obtained at a window size of 21 (Figure 1a). Therefore, we have continued with the rest of the experiments using this window size. Sequence windows (or 21mers) containing neddylation sites were referred in short as positive windows and the rest as negative windows.

### Properties of neddylation sites

Neddylation site properties have not been investigated thoroughly before. In order to fill this gap, we have performed statistical analyses to pinpoint the differences between neddylation sites and other lysine centred sequence windows. Initially, we have investigated differences in amino acid preferences at each position of a sequence window. We have performed two separate analyses for this case: two-sample sequence logo analysis and amino acid enrichment analysis using chi-square test of independence. For both of these analyses, all sites from training, validation and test sets were combined to reveal the characteristics of neddylation sites using all available data.

**Figure 2:**
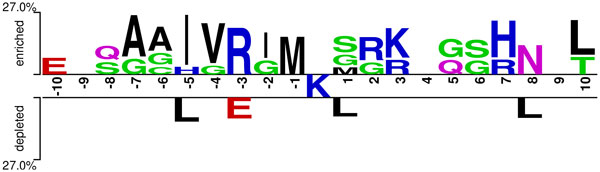
**Sequence properties of neddylation sites in complete dataset**. Sequence logo of lysine-centred windows, showing enrichment and depletion of amino acids in particular positions. This logo was created using Two-Sample Logos [9] with default parameters.

Two-sample logo analysis [9] with default parameters showed that immediate vicinity of central lysine was enriched with a methionine residue and glycine residues (Figure 2). A band of positively charged amino acids was enriched in the close positions of central lysine while even further positions were enriched with polar amino acids, glycine residues, and aliphatic amino acids in the upstream.

Statistical testing results with Bonferroni correction validated the methionine finding of two-sample logo analysis. Methionine enrichment at position -1 was found to be statistically significant, *χ*^2 ^ (1, N = 1082) = 49.63, p < 10^-8^. Methionine at position -1 was present in 20% of the neddylation sites while only 2% of non-neddylated sites had a methionine residue at this position. Similarly, isoleucine presence was also significantly different in position -5 of neddylated (present in 29%) and non-neddylated (present in 7%) sites, *χ*^2 ^ (1, N = 1082) = 35.72, p < 10^-5^. As it was observed in sequence logo, significantly more valine residues in position -4 were present in neddylated sites (25%) than non-neddylated sites (7%), *χ*^2 ^ (1, N = 1082) = 22.51, p = 0.002. Another noteworthy difference, the frequency of arginine at position -3 of positive windows was found to be different than that of negative windows, *χ*^2 ^ (1, N = 1082) = 45.01, p < 10^-7^. It was observed that neddylation sites have positively charged amino acids at this position more frequently (25% of positive windows, 4% of negative windows). All of the listed amino acid preference differences were partly identified before for determining a consensus cullin neddylation motif ([IL][VIT][RQ][IS][MLV]**K**[MAS][RHE]) [10]. Our findings support this motif for aforementioned positions. However, this motif does not account for other amino acid presence differences that were observed in this dataset, such as over-representation of histidine residues in position +7 of neddylation sites (p < 10^-7^), and over-representation of asparagine in position +8 in neddylation sites (p = 0.004).

Position-independent aspartic acid (D), and glutamic acid (E) counts in a window were also found to be significantly lower in positive windows than negative windows, p < 10^-4^. This finding was in parallel with positive amino acid enrichments, suggesting a preference of positive charges instead of negative ones in the vicinity of lysine residues in neddylation sites. Besides, ratio of average occurrence of aspartic or glutamic acid residues in a window to average occurrence of these residues in whole protein indicated that these residues are significantly less observed in neddylated sites (p < 0.0002).

Apart from amino acid preferences, we have identified various significant physicochemical differences between positive and negative windows. Positive windows were found to be flexible more frequently (39% of positive windows, 12% of negative windows) than negative windows, *χ*^2 ^ (1, N = 1082) = 31.96, p < 10^-4^. Parallel to this finding, flexibility confidence ratio-based measure (ConfRat) was also found to be significantly different, *χ*^2 ^ (1, N = 1082) = 21.59, p = 0.004. 43% of positive windows had more confidently predicted flexible residues than rigid ones while it was only 17% for the negative windows. Functionally similar to window flexibility, window disorder predictions with a cut-off of 0.5 were also found to be different between positive and negative windows, *χ*^2 ^ (1, N = 1082) = 54.80, p < 10^-9^. Again, 41% of positive windows were found to be disordered while only 9% of negative windows had this feature. We have also investigated the co-occurrence of window flexibility and window disorder predictions by further looking into sequence windows that have at least one these two properties. We found that majority of positive windows that were predicted to be flexible were also predicted to be disordered (19 sites). Only a single site was predicted to be flexible but not disordered. Also, two sites were predicted as disordered but not flexible. This was not the case for negative windows. Results indicated that these negative windows were a mixture of flexible but not disordered (66 windows), disordered but not flexible (35 windows), and both flexible and disordered (56 windows).

Lastly, positive windows were found more frequently at the N- or C- terminus of the protein than negative windows, *χ*^2 ^ (1, N = 1082) = 41.22, p < 10^-6^. 57% of the positive sites were found to be at the termini while only 19% of the negative sites were in this region. 10% of the all residues located near N- or C- terminus were considered as at "termini" for this calculation. Details can be found in the methods section. Statistical significance of this property was also sensible as termini regions are relatively free to interact with partners. Complete list of statistically significant features can be found in supplementary information (Additional File 2, Table S1).

### Model performance and feature selection

Incremental feature selection using the mRMR ordered feature list resulted in an optimal feature set containing 49 features (Figure 1b). This feature set included evolutionary conservation scores of some positions, both grouped and non-grouped amino acid preferences at some positions, average occurrence ratios and occurrence counts of particular amino acids or amino acid groups, binary window disorder prediction, and hydrophobicity of particular positions in the window. An interesting result was that solvent accessibility prediction and window flexibility predictions were not present in the retained feature set. Top 10 of the selected features can be found in Table 1 and the complete list was given in supplementary information (Additional File 3, Table S2). Additionally, contributions to prediction performance of each related feature set in the selected features were evaluated by removing each set one by one from the training dataset (Table 2). These results showed that positional amino acid preferences -or sequence motifs- are the most contributing features in prediction.

**Table 1:** Top 10 of the selected features.

Order	Feature	Position
1	M presence	-1
2	PSSM score of K	-7
3	I/V/L/M presence	+8
4	Termini	-
5	D/E occurrence count	-
6	R presence	-3
7	I presence	-5
8	Hydrophobicity	-2
9	A presence	-7
10	V presence	-4

**Table 2:** Effect of different feature sets in selected features on prediction performance.

	**5-fold stratified cross-validation**^†^				
**Information**	**Acc**	**Sp**	**Sn**	**MCC**	**AUC**

All selected features	0.91	0.91	0.75	0.44	0.95
without amino acid preferences	0.88	0.89	0.63	0.33	0.88
without amino acid preferences (grouped)	0.89	0.90	0.65	0.35	0.91
without disorder	0.89	0.90	0.72	0.40	0.93
without termini	0.91	0.93	0.68	0.42	0.94
without amino acid occurrence counts	0.91	0.91	0.74	0.44	0.94
without hydrophobicity features	0.91	0.91	0.75	0.44	0.94
without amino acid occurrence ratios	0.91	0.92	0.72	0.43	0.94
without PSSM features	0.91	0.92	0.74	0.44	0.94

^† ^Cross-validation (CV) results were reported as means of 100 repeats. As two standard errors were not exceeding 0.01, they were not reported.

Using all 49 selected features, we have further analysed the effect of class weights on classification accuracy. We observed that the average AUC increases with the weight assigned to positive instances and becomes almost constant after 1:15 (Additional File 4, Figure S2). Therefore, optimal class weight was selected as 1:15.

**Figure 3:**
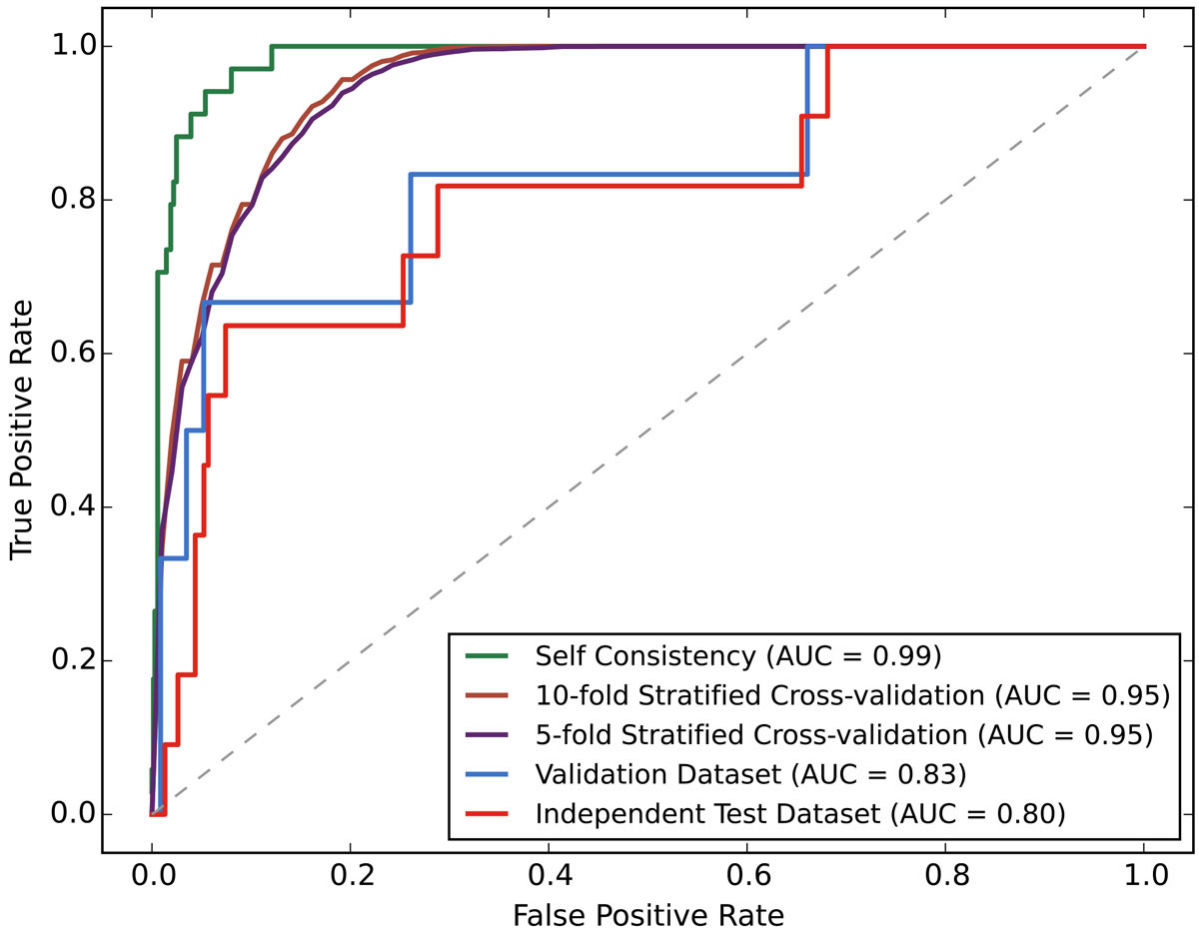
**ROC curves**. ROC curves of classification model with different evaluation strategies.

After optimization of class weights and features, we have investigated training performance of our model using different evaluation strategies (Table 3). Mostly due to the effect of the high weight assigned to the positive class, our model learned positive instances perfectly, as shown in the self-consistency results. However, this was not an overlearning case, considering consistently high specificity and sensitivity in cross-validation results. We also plotted receiver-operating-characteristic (ROC) curves to further investigate the behaviour of our prediction model (Figure 3). Steadily high AUC values suggest that our model demonstrates a robust performance. Besides, independent test set results indicate that the high performance of our model was not limited to the training data, as holdout test data results show an AUC of 0.80.

**Table 3:** Performance of classification model under different evaluation strategies.

Evaluation Strategy	Acc	Sp	Sn	MCC	AUC
Self-consistency	0.92	0.92	0.94	0.56	**0.98**
5-fold stratified cross-validation^†^	0.91	0.91	0.75	0.44	**0.95**
10-fold stratified cross-validation^†^	0.91	0.91	0.76	0.45	**0.95**

Validation set	0.90	0.91	0.67	0.39	**0.83**

Holdout set	0.90	0.91	0.64	0.35	**0.80**

^† ^Cross-validation (CV) results were reported as means of 100 repeats. As two standard errors were not exceeding 0.01, they were not reported.

### Comparison with known and derived motifs

Our model is the first ever neddylation site prediction model in literature. Therefore, it is not possible to compare with a state of the art method. In order to overcome this problem, we have used 21mers to discover neddylation motifs using MEME [11]. MEME was used in two different modes: normal and discriminative mode. Using normal mode with one occurrence per sequence and minimum motif width of 3, MEME identified a single possible motif (AAIV[RQ]IM**K**S) with e-value of 3.1x10^-7^. Using discriminative mode with same parameters, MEME gave a similar result. It identified a single motif (IVRIM**K**S) with e-value of 3.8x10^-5^. These two motifs and one consensus cullin neddylation motif ([IL][VIT][RQ][IS][MLV]**K**[MAS][RHE]) [10] in literature were used for comparison (Table 4). Test set performance of these motifs revealed that they can correctly predict only a single CUL3A neddylation site in *A. thaliana *and misclassify all non-cullin neddylation sites. Additionally, another cullin neddylation site was misclassified by all three motifs. Performance evaluation of all motifs indicates that there is no generalizable neddylation motif possible with the data available in the literature. Therefore, it can be argued that sequence by itself is not sufficient in capturing variability of neddylation sites and additional sequence characteristics are needed for predicting neddylation sites in both cullins and other proteins.

**Table 4:** Comparison of SVM prediction performance to known or predicted motifs using test set.

Predictor	Acc	Sp	Sn	MCC
AAIV[RQ]IMKS^1^	0.96	1.00	0.09	0.30
IVRIMKS^2^	0.96	1.00	0.09	0.30
[IL][VIT][RQ][IS][MLV]K[MAS][RHE]^3^	0.95	1.00	0.09	0.20

SVM	0.90	0.91	**0.64**	**0.35**

^1^This motif was derived using MEME normal mode. ^2^This motif was derived using MEME discriminative mode. For prediction of both motifs, same training sites with SVM training were used. ^3 ^This motif was reviewed in [10].

### Investigation of multiple ubiquitin-like modifications at same target sites

Ubiquitylation and neddylation have been previously shown to act together to coordinate target protein activity [12]. Also, some proteins are shown to be modified with multiple ubiquitin-like modifiers simultaneously [13-17]. This may be due to dynamic control of cellular signaling pathways, similarity of Ubl conjugation mechanisms, lack of specificity of E2 enzymes, and enzyme sharing under certain circumstances [18]. In order to identify possible sites with multiple Ubl modifications, we have obtained all known sumoylation and ubiquitylation sites of *A. thaliana, R. norvegicus, M. musculus*, and *S. cerevisiae *proteins from dbPTM [19] and applied our prediction method. Summary of obtained dataset can be found in supplementary information (Additional File 5, Table S3). We have predicted putative neddylation sites in these proteins with two different thresholds: medium (threshold = 0) and high (threshold = 1). These thresholds were set to decision values outputted by SVM, which represent the distance of the samples to the separating hyperplane. Higher absolute decision values imply deeper points or more confident decisions. Performance metrics under different thresholds can be found in supplementary information (Additional File 6, Table S4).

**Figure 4:**
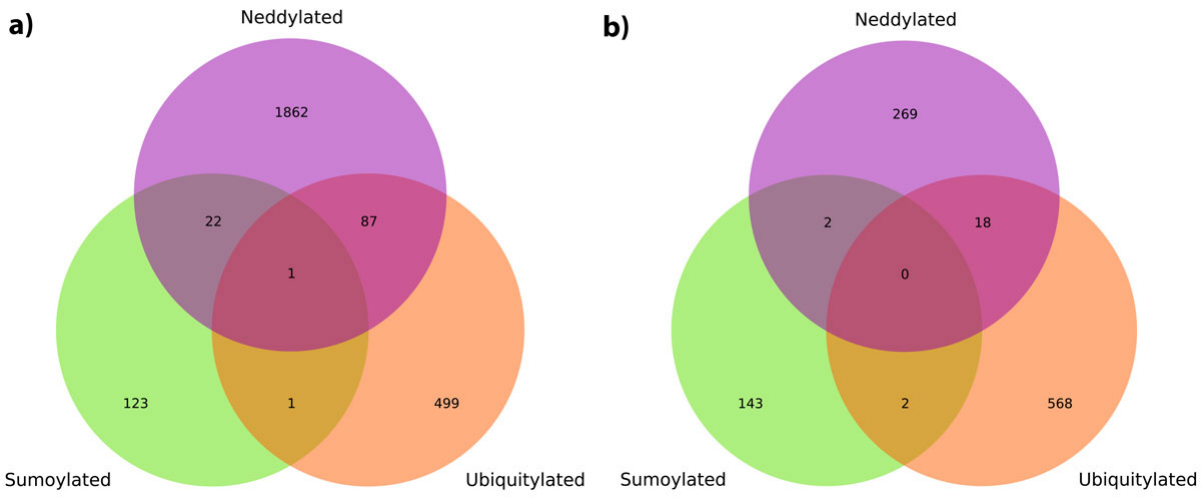
**Venn diagrams of shared modification target sites between predicted neddylation sites and known sumoylation and ubiquitylation sites**. Experimentally identified sumoylation and ubiquitylation sites were obtained from dbPTM [19]. Possible neddylation sites of these proteins were then predicted with neddylation prediction method. Number of common sumoylation, ubiquitylation and neddylation sites using a) medium and b) high neddylation prediction thresholds were reported in the diagram.

Medium threshold prediction results show high amounts of neddylation sites in proteins obtained from dbPTM (Figure 4a). 326 proteins out of 358 were predicted to have at least one neddylation site (minimum: 1, maximum: 35, median: 5 sites) under medium threshold. Large number of predicted neddylation sites may indicate an over-prediction of model, since it was developed with a small dataset. However, mouse IκBα protein were predicted to be sumoylated, ubiquitylated and neddylated at position 21. This prediction was noteworthy as it may indicate a highly dynamic control of NF-κB pathway.

Using more confident neddylation predictions with high threshold, neddylation predictions dropped significantly (Figure 4b). 164 proteins out of 358 were predicted to have at least one predicted neddylation site (minimum: 1, maximum: 8, median: 1 site). Additionally, we have failed to observe any site that is modified by all three of the modifications. Species-specific results can be found in supplementary information (Additional File 7, Figure S3a-h).

In addition to using our predictor with known other Ubl modification sites, we have used other Ubl prediction methods on known neddylation sites to determine possible unknown shared sites. We have merged training, validation and test sets of our method and predicted their Ubl modification status with a ubiquitylation predictor, UbPred [20], and a sumoylation predictor, GPS-SUMO [21]. For sumoylation site predictions, only covalent attachment of SUMO proteins were considered, and non-covalent SUMO interaction predictions were discarded.

UbPred predicted ~20% (10/51), ~12% (6/51), and ~2% (1/51) of the neddylation sites as ubiquitylation with low, medium and high confidence respectively. UbPred training dataset was not searched for presence of known neddylation sites, as Uniprot IDs and site locations were not reported in original publication of UbPred. On the other hand, GPS-SUMO predicted ~6% (3/51), ~4% (2/51), and ~4% (2/51) of neddylation sites as sumoylated with low, medium and high thresholds respectively. None of the predicted sites were present as sumoylation sites in GPS-SUMO training dataset. None of the sites predicted by UbPred and GPS-SUMO were the same site.

Prediction difference between UbPred and GPS-SUMO was meaningful, since it has been observed that in environments with elevated NEDD8 to ubiquitin ratio, neddylation is dependent on ubiquitylation enzymes [18]. Therefore, it is possible that UbPred would capture targets of shared enzymes under certain circumstances. Lastly, difference of predicted sites and low prediction ratio in known neddylation sites indicate that neddylation sites have their unique patterns; therefore, in silico identification of neddylation sites require separate predictors.

In conclusion, our analyses support the findings on multiple ubiquitin-like modifications at the same target site. However, it is not possible to identify intricate activation or repression relationship between different type of Ubl modifications using our findings.

## Discussion

Neddylation is one of the vital mechanisms in cellular machinery, and it significantly alters the fate of any target protein. Hence, as with other post-translational modifications, it is one of the key components in understanding the function, regulation and lifetime of a protein. Besides, aberrations in neddylation or deneddylation of a target protein may result in many anomalies and diseases. Therefore, in addition to guiding proteomic studies, *in silico *identification of neddylation sites may be of help in functionalizing disease-related genomic variants and understanding aetiology and pathogenesis of many diseases, such as Alzheimer's and Parkinson's. This paper introduces the first method for predicting neddylation sites from protein sequences that may aid in such studies.

Conformational flexibility was previously hypothesized to be a general feature of target sites in sumoylation and more generally ubiquitin-like protein modification mechanisms [22]. We can extend this hypothesis to include disorder, and more generally, freedom of movement of target sites. In this study, we showed that neddylated and non-neddylated sites are significantly different in conformational flexibility and disorder. Also, disorder is one of the most important factors in prediction of neddylation sites. We have demonstrated this for the first time in literature for neddylation site prediction. Besides, this finding is noteworthy as freedom of movement provides easy adjustment of partners in E2/E3-target protein interaction pairs. It also implies that structural information may improve neddylation site prediction; however, sequence-based structural information predictors are sufficient for such purposes. Another interesting factor related to the movement freedom of the target site is the site's location. We observed that many of the neddylation sites are actually located in the C- or N- terminus. The statistical significance of all features related to freedom of movement (flexibility: p < 10^-4^, disorder: p < 10^-9^, termini: p < 10^-6^) supports our hypothesis, in which Ubl target site recognition requires a certain freedom of movement. Furthermore, this finding is not limited to only neddylation sites, but can be employed in the development of various other post-translational modification prediction methods, such as ubiquitylation and phosphorylation. Previously, we have exploited conformational flexibility and disorder in sumoylation site prediction with a different strategy, in which we were interested in conformational flexibility and disorder of the central lysine only [23]. In contrast, we used average window conformational flexibility and disorder in this work. Window-wise measures of flexibility and disorder are more informative than measures of the central lysine, as site recognition is more likely to be based on not only the target lysine residue, but also the amino acids in the vicinity of the target residue.

Moreover, we have investigated neddylation site properties to assist understanding the regulation of neddylation and the mechanisms of site recognition in a large scale. Among other amino acid preferences, methionine enrichment at position -1 was interesting since sumoylation consensus site motif ([IVLM]**K**x[DE]) includes a methionine residue at the same position. On the other hand, this shared enrichment does not indicate that sumoylation and neddylation sites have common overall patterns. For instance, while sumoylation sites are enriched with acidic amino acids after the central lysine, neddylation sites tend to have more basic amino acids. Therefore, these two post-translational modifications may require separate mechanisms for target recognition. Additionally, we also observed that some of the positions are occupied with conserved residues in neddylation sites, as three PSSM scores were retained in feature selection. One of PSSM scores (K at position -7) was ranked second in the ordered feature list.

The major limitation of this study was the size of the dataset used. The discovery of neddylation is relatively recent and studies on neddylation target proteins have been limited so far. This limitation may influence both the prediction performance and the confidence of statistical analyses. For instance, some of the statistically significant features may be declared significant only due to frequency differences particular to this dataset, not to an underlying biological principle. However, the ever-increasing amount of experimentally validated neddylation target sites as well as further studies on neddylation site recognition may improve prediction performance. Another performance limiting factor can be mediated post-translational modifications. It has been argued that in some of the Ubl modifications, special proteins, called degrons, mediate substrate site recognition with E3 enzymes [24]. Alternatively, neddylation of a protein may require another post-translational modification to reveal its neddylation site. Such cases would be completely missed out by our method and most of the other Ubl target site predictors. Another issue would be the reliability of the dataset as neddylation also uses ubiquitylation enzymes under elevated free NEDD8 to ubiquitin ratio conditions [18]. Therefore, there is still a need for experimental studies on reliable neddylation sites and various aspects of neddylation site recognition mechanisms.

## Conclusions

In conclusion, we have developed a novel neddylation site prediction method, exploiting the sequence characteristics and sequence-derived features of residues located in the vicinity of lysines. Additionally, we have investigated the frequency of these properties to reveal valuable information for further understanding neddylation. Major direction for future work lays in the development of a web service for neddylation site prediction to make it accessible to the biological research community.

## Methods

### Dataset preparation

We have searched PubMed with keywords "nedd8", "neddylation", "nedylation", "rub1", "rub2", "rub3", and "rubylation", and manually collected 65 sites in 31 proteins from ~680 articles, published before 1 February 2015.

Among manually collected sites, 6 were discarded due to neddylation being shown only *in vitro*, and 3 were discarded as neddylation was not reported in a single amino acid resolution. After this elimination, primary sequences of 30 proteins were retrieved from UniProt [25]. Redundant protein sequences in this set were eliminated using CD-HIT [26]. CD-HIT clusters sequence sets and selects a representative sequence of each cluster that meets an identity threshold. We have clustered sequences with 0.4 threshold, so that no two sequences sharing a sequence identity >40% were left in the dataset. After such an elimination procedure, dataset was left with 25 proteins and 51 sites (Additional File 8, Table S5). Remaining lysine residues that have no evidence of neddylation in these proteins (1031 sites) was used as negative samples.

Samples from each class were randomly distributed into train and holdout sets with a ratio of 2:1. Holdout set was further randomly divided into validation and independent test sets with a ratio of 1:2. After this distribution, training set was formed of 34 positive sites and 687 negative sites, while validation set was formed of 6 positive sites and 115 negative sites and independent test set was formed of 11 positive sites and 229 negative sites. All sites in the training, validation, and indepentent test datasets can be found in the Bitbucket repository of this article (see "Availability of supporting data" section).

### Feature construction

We prepared a dataset for analysis by defining sequence windows around the central lysine residues flanked by n residues upstream and n residues downstream, forming a 2n+1 amino acid long sequence segments. All sequence windows that contain experimentally identified neddylation sites were considered as the positive set. The rest of the sequence windows were assumed to be non-neddylated and used as the negative set. In order to find the optimal window length, we have tried various values from 5 to 27 and measured classification performance.

#### Sequence encoding

In order to exploit possible sequence motifs, each position in the sequence windows can be represented by a 20-dimensional vector, where each dimension indicates an amino acid and only one dimension may contain a value of 1. In case of missing positions, all corresponding amino acid vectors were left as zeros. We have also grouped amino acids according to Sezerman grouping (Additional File 9, Table S6) to capture common biochemical properties together. This grouping may help identify amino acids that are rare by themselves, but when grouped, they become frequent additively. For each position in the sequence window, this information was represented by an 11-dimensional vector, and the same strategy was followed with non-grouped amino acids.

Additionally, we have used position independent amino acid composition of a window. We have calculated two feature sets for this purpose: occurrence counts of amino acids, and ratio of average occurance of amino acids in the window to average occurance in the whole protein. Each feature set was represented with 20-dimensional vector for non-grouped amino acids and 11-dimensional vector for Sezerman grouped amino acids.

#### Evolutionary conservation

Evolutionary conservation of a residue often indicates an importance in biological function. If a particular residue is conserved, it may be located in a functionally important site, like an active site or a post-translational modification target site. Position-specific iterative BLAST (PSI-BLAST) can identify conserved residues and create position-specific scoring matrices (PSSMs) [27]. Each position in a sequence window was represented by 20-dimensional vector indicating probability of conservation of amino acids. Protein sequences were searched against nr database (-db nr) using PSI-BLAST program from BLAST+ toolkit (version 2.2.26+) with three iterations (-num_iterations 3), and inclusion e-value threshold of 1e-5 (-inclusion_ethresh 1e-5). A high score found in PSSM would then reflect a strong conservation of amino acid in that particular position and indicate a possible role in neddylation site recognition.

#### Secondary structure and solvent accessibility

Structural constraints are argued to be one of the determining factors in lysine selectivity for ubiquitylation and other ubiquitin-like protein modifications [24]. Among these constraints, secondary structure may affect whether a site can be recognized. Examples of this phenomenon can be found in other ubiquitin-like protein modifications such as sumoylation [28]. UBC9, a sumoylation E2 enzyme, cannot recognize sumoylation consensus sites in stable helical structures [29]. In order to explore similar effects; we have introduced a secondary structure feature to the dataset. Secondary structure predictions were performed using PSI-PRED web server (version 3.3) with default settings [30,31]. Secondary structure of central lysine residue was represented by a 3-dimensional binary vector.

Additionally, we have introduced a measure to investigate whether central lysine residue was located in the C- or N- terminus of the secondary structure element. We have declared residues to be in the secondary structure termini, if they were one of the amino acids residing in the 15% from both ends of that particular secondary structure element.

Solvent accessibility may also be considered one of the key factors in determining whether a lysine residue is accessible for recognition. In order to capture this information we have employed binary solvent accessibility predictions of central lysine using WESA tool [32].

#### Conformational flexibility and disorder

We hypothesize that the lysine residue in the neddylation site should have conformational flexibility to be recognized by E2 or E3 enzymes in the neddylation pathway. Supporting this hypothesis, it has been previously shown that flexible central lysine residues were more frequent in sumoylation target sites [23]. In order to obtain conformational flexibility information, we have used FlexPred in PSSM-based encoding mode, which mainly predicts residue positions involved in conformational switches [33]. FlexPred takes protein sequences as input, predicts whether each residue in that protein is flexible or rigid, and provides a confidence value of its predictions. We have calculated a binary feature representing overall flexibility of sequence window, determined by the majority of amino acids in the window. Additionally, we have created a binary value confidence-based flexibility feature aimed to represent the confidence in the whole window flexibility. For every sequence window w_i_, this feature was calculated using the confidence values of amino acid flexibility predictions in that particular sequence window. The calculation was performed according to the following formula:

(1) $$\text{ConfRat}(w_{i})=\left\{\begin{array}{cc} 1 & \frac{\sum Confidence_{Flexible}}{\sum Confidence_{Rigid}}\geq 1\\ 0 & otherwise \end{array}\right)$$

In addition, we have used disorder predictions to investigate the effect of intrinsically unstructured regions that may not be predicted as flexible. Previously, it has been shown that anaphase promoting complex/cyclosome (APC/C) ubiquitin ligase substrates are enriched in disordered regions [34]. Similarly, predicted disordered regions occur more often in sumoylation target sites [23]. Therefore, we have used disorder predictions by IUPred [35]. IUPred predicts disorder from amino acid sequences by estimating the capacity to form stabilizing contacts of polypeptides and yields a disorder tendency value for each amino acid [35]. We have converted this information into two separate window-wise disorder measures. The first one was created by average disorder tendencies of the amino acids located in that sequence window. The second measure was created by setting a threshold of 0.5 for the average disorder tendency and forming a binary feature. Sequence windows were declared as disordered if their average disorder tendency was larger than or equal to 0.5, and ordered otherwise.

#### Hydrophobicity and amino acid volumes

The efficacy of using hydrophobicity in ubiquitin-like post-translational modification site prediction was shown in various publications before [23,36]. However, most of the methods involve hydrophobicity in a residue-wise fashion. In this work, we have used Hopp & Woods hydrophobicity scale [37] to calculate average hydrophobicity of a sequence window. This scale was selected as our previous work indicated that it performs better for our classification task [23]. Using average hydrophobicity of sequence windows was an intuitive choice, as hydrophobic or hydrophilic patches were more likely to be identified by an average based approach instead of a residue-wise one. In addition, we have introduced average hydrophobicity of pre- and post-lysine sub-windows as two separate features to observe if any sub-window specific hydrophobicity patterns exist.

Moreover, we have estimated the volume of pre- and post-lysine sub-windows using Kharakoz's estimated amino acid volumes [38]. The volume of each sub-window and the difference between the two sub-windows added three different features to the dataset. These features were introduced as they may represent the accessibility for recognition of the central lysine residue.

#### Other features

Several custom features were constructed to capture various sequence properties, such as location of the central lysine residue of a window. We have constructed a binary vector indicating whether the central lysine of the sequence window located within the 10% of the N- and C- termini. In order to take the sequence length of the protein into account, we have created another measure, which has a value of 1*sequence lenth, if the central lysine is located at the termini, and +1* sequence length if it is not.

### Statistical testing

Statistical significance of all features was assessed using chi-square test of independence for binary features and Mann-Whitney U test for continuous valued features. Bonferroni correction has been applied for multiple testing correction. All p-values have been adjusted according to this procedure.

All statistical tests were performed using Python (version 2.7.5, The Python Consortium; http://www.python.org), with the SciPy library (version 0.11.0, The Scipy Consortium; http://www.scipy.org).

### Feature selection

Feature selection was performed in a two-stage strategy. The first step was the ordering of features according to minimal-redundancy-maximal-relevance (mRMR) criterion [39,40]. Afterwards, using the ordered feature list, dimensionality is reduced by incremental feature selection.

#### mRMR criterion

mRMR criterion aims to select a feature subset that represents the statistical property of the target classification variable. Selected features are aimed to be most dissimilar to each other and most similar to the classification variable [39]. Mutual information is used in this method to determine the "relevance" and "redundancy" of features. mRMR software provides two lists as output: an ordered list of features by relevance (MaxRel), and an ordered list of features by both relevance and redundancy (mRMR Features). We have used mRMR software with a discretization threshold of 1 (-t 1), and a maximum number of features of 50 considering the size of our dataset. Other parameters were left as default.

#### Incremental feature selection

In the ordered mRMR list, higher ranked features are better than lower ones. However, order information by itself is not sufficient in determining the features to be retained. Therefore, selecting the optimal feature subset is another issue. Finding an optimal subset of features can be possible by incrementally adding features from the highest scored feature to the lowest in the mRMR list and evaluating the performance of the resulting classifier. In this work, we have employed this strategy and trained classifier models using fine-tuned support vector machines. Average area under receiver-operator-characteristic curve (AUC) of 100 repeats of 5-fold stratified cross validation was used as a performance measure in determining the number of features to be retained (for details see next subsection).

### Model building and performance assessment

We have used support vector machines to build classification models and predict neddylation sites from protein sequences. Support vector machine (SVM) is a common machine learning algorithm that tries to optimize separation between classes using a hyperplane in a high-dimensional space. SVMs may be used for classification, regression or other tasks in various domains. However, in spite of their advantages, SVMs require fine-tuning of multiple parameters.

In this work, we have used LibSVM [41] implementation of SVMs via a wrapper provided in scikit-learn Python module [42]. In order to fine-tune classification models, we have performed grid searches for *C *and *g *parameters of the radial basis kernel. The optimal *C *parameter was searched from 2^-5 ^to 2^15 ^by doubling the parameter in each iteration and gamma (*g*) parameter was searched from 2^-15 ^to 2^3 ^with the same strategy.

Shortcomings of SVMs include their sensitivity to class imbalance, resulting in a degraded classification performance of the minority class. In order to overcome this problem, we have exploited the class weights parameter, which sets the C parameter of a SVM kernel to C*weight. We have investigated the effect of various class weights (1:1, 1:5, 1:10, 1:15, 1:20, 1:25, and 1:30) on finding the optimal classification model.

The performance of models was assessed using various strategies: self-consistency test, 5-fold stratified cross-validation, and 10-fold stratified cross-validation. Self-consistency test was performed with training dataset used as both training and test dataset. Stratified cross-validation was selected over normal cross-validation since we have observed folds that do not contain any positive instances in some repeats. In order to perform stratified cross-validation, data was divided into k-folds, while preserving the class balance in each fold. We have used StratifiedKFold function in scikit-learn library for this purposes. This function assigns each sample to a test fold index using individual k-fold splitting for each class to respect the class balance. 5-fold and 10-fold stratified cross-validations are repeated 100 times and mean values of evaluation measures were reported. In order to assess the classification performance, we have calculated classification accuracy (Acc), specificity (Sp), sensitivity (Sn), Matthew's correlation coefficient (MCC), and area under receiver-operating-characteristic curve (AUC). Details of the performance measures can be found in the Additional File 10. AUC was used as a main performance measure for determining the classification performance. This measure indicates the extent of the discriminatory power of a classifier at different operating points. AUC values approaching to 1 are considered to be good performance while an AUC value of 0.5 represents a random model. Additionally, whenever the same AUC was observed for two compared models, the tie was broken according to validation set sensitivity. Sensitivity of validation set was selected as a secondary measure, due to small number of positive sites in this dataset. In rare occasions, when these two measure did not break the tie, specificity of validation set was used.

## Availability of supporting data

The data sets used in this article and the standalone version of neddylation site prediction method developed in this article are available in a Bitbucket repository at https://bitbucket.org/asyavuz/neddypreddy.

The data supporting the results of this article are included within the article and its additional files.

## List of abbreviations used

PTM: post-translational modification; Ubl: ubiquitin-like modifier; SVM: support vector machine; Acc: accuracy; Sp: specificity; Sn: sensitivity; MCC: Matthew's correlation coefficient; ROC: receiver operator characteristics; AUC: area under the ROC curve.

## Competing interests

The authors declare that they have no competing interests.

## Authors' contributions

ASY participated in the design of the study, carried out the experiments, performed the statistical analyses, and drafted the manuscript. NBS collected the dataset and carried out the experiments. OUS conceived the study, and participated in its design and coordination, and helped to draft the manuscript. All authors read and approved the final manuscript.

## Supplementary Material

Additional file 1 Figure S1 (*.pdf). Diagram of neddylation pathway. Click here for file

Additional file 2 Table S1 (*.pdf). Complete statistical testing results. Click here for file

Additional file 3 Table S2 (*.pdf). Complete list of features after two-staged feature selection using mRMR and incremental feature selection. Click here for file

Additional file 4 Figure S2 (*.pdf). Mean classification AUC using different class weights for SVM training. Click here for file

Additional file 5 Table S3 (*.pdf). Summary of post-translational modification sites obtained from dbPTM. Click here for file

Additional file 6 Table S4 (*.pdf). Validation set performance under different decision thresholds. Click here for file

Additional file 7 Figure S3 (*.pdf). Species-specific Venn diagrams showing the number of sites modified by multiple ubiquitin-like modifications at the same site. Click here for file

Additional file 8 Table S5 (*.pdf). Complete list of neddylated sites utilized in this study. Click here for file

Additional file 9 Table S6 (*.pdf). Sezerman grouping of amino acids. Click here for file

Additional file 10 Supplementary Information (*.pdf). Supplementary methods can be found in this file. Click here for file

## Declarations

Publication charges for this article have been funded by the corresponding author.

This article has been published as part of *BMC Bioinformatics *Volume 16 Supplement 18, 2015: Joint 26th Genome Informatics Workshop and 14th International Conference on Bioinformatics: Bioinformatics. The full contents of the supplement are available online at http://www.biomedcentral.com/bmcbioinformatics/supplements/16/S18.
