# Supplementary material for: Prediction of neddylation sites from protein sequences and sequence-derived properties
Source: BMC Bioinformatics. 2015 Dec 9;16(Suppl 18):S9. doi: 10.1186/1471-2105-16-S18-S9 (PMC4682398; doi:10.1186/1471-2105-16-S18-S9)
Supplement: Additional file 1 — Figure S1 (*.pdf). Diagram of neddylation pathway. [file 1471-2105-16-S18-S9-S1.pdf]

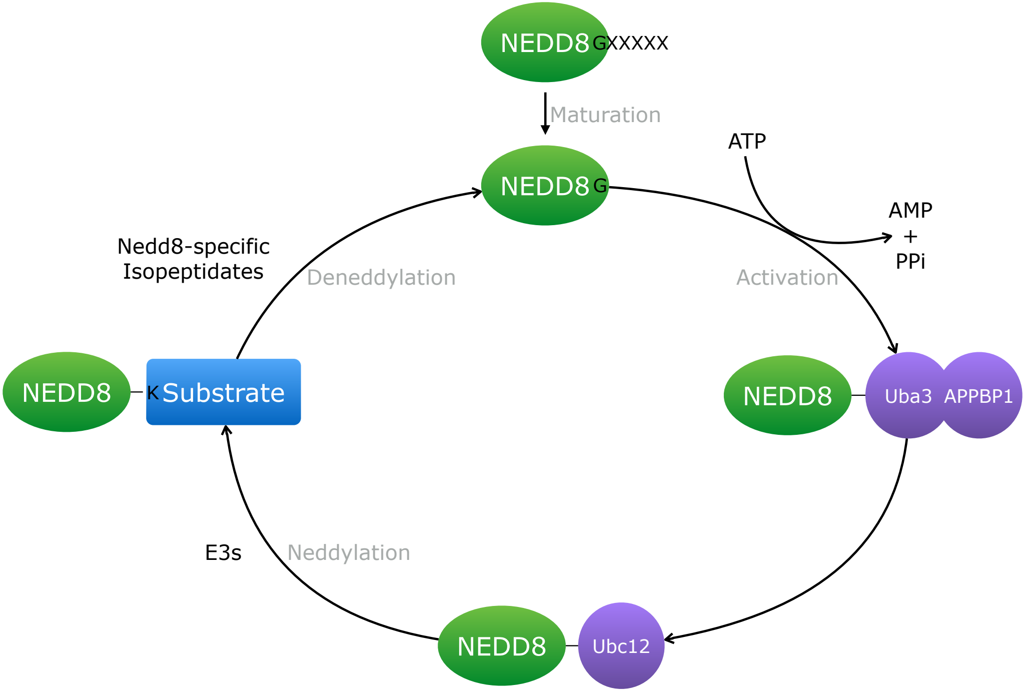

Fig. S1. Neddylation pathway consists of 4 steps. Newly synthesized NEDD8 protein is matured after cleavage of an invariant peptide located after Gly76. Afterwards, matured NEDD8 protein is activated using an ATP and bound to an E1 enzyme, Uba3-APPBP1 heterodimer. NEDD8 is then transferred to an E2 enzyme, Ubc12. With the help of E3 enzymes, Ubc12 transfers NEDD8 to the target site. After its function is completed, attached NEDD8 residue may be cleaved by NEDD-8 specific isopeptidades and released for further use.
