# Supplementary material for: Prediction of neddylation sites from protein sequences and sequence-derived properties
Source: BMC Bioinformatics. 2015 Dec 9;16(Suppl 18):S9. doi: 10.1186/1471-2105-16-S18-S9 (PMC4682398; doi:10.1186/1471-2105-16-S18-S9)
Supplement: Additional file 2 — Table S1 (*.pdf). Complete statistical testing results. [file 1471-2105-16-S18-S9-S2.pdf]

Table S1. Statistical testing results.

Feature	Test	Test Statistic	Positive Ratio/Mean	Negative Ratio/Mean	p-value
Termini (Sequence Length)	Mann-Whitney U	9882	-155.18 (SD: 524.44)	443.54 (SD: 584.14)	5.33E-11
Window Disorder (Binary)	Chi-square	54.80	0.41	0.09	1.49E-10
M presence at position -1	Chi-square	49.63	0.2	0.02	2.08E-09
R presence at position -3	Chi-square	45.01	0.25	0.04	2.20E-08
H presence at position +7	Chi-square	44.11	0.2	0.03	3.48E-08
Termini	Chi-square	41.22	0.57	0.19	1.52E-07
I presence at position-5	Chi-square	35.72	0.29	0.07	2.55E-06
Window Flexibility	Chi-square	31.96	0.39	0.12	1.76E-05
Occurence count of D or E in a window	Mann-Whitney U	14413	1.65 (SD: 1.47)	2.82 (SD: 1.55)	3.00E-05
R/K/H presence at position -3	Chi-square	29.37	0.43	0.15	6.69E-05
Average occurance ratio of D/E	Mann-Whitney U	14786	0.58 (SD: 0.45)	0.98 (SD: 0.54)	1.43E-04
Window Disorder (Real)	Mann-Whitney U	15259	0.42 (SD: 0.24)	0.25 (SD: 0.17)	4.59E-04
A presence at position -7	Chi-square	25.60	0.25	0.06	4.70E-04
A presence at position -7	Chi-square	25.60	0.25	0.06	4.70E-04
V presence at -4	Chi-square	22.51	0.25	0.07	2.34E-03
Window Flexibility (ConfRat)	Chi-square	21.59	0.43	0.17	3.78E-03
N presence at position +8	Chi-square	21.30	0.18	0.04	4.38E-03
Occurence count of Y or F in a window	Mann-Whitney U	16589	1.02 (SD: 1.64)	1.64 (SD: 1.30)	4.88E-03
R/K/H presence at position +7	Chi-square	19.79	0.37	0.14	9.68E-03
Occurence count of E in a window	Mann-Whitney U	17390	1.00 (SD: 1.07)	1.72 (SD: 1.29)	2.70E-02
