# Supplementary material for: Prediction of neddylation sites from protein sequences and sequence-derived properties
Source: BMC Bioinformatics. 2015 Dec 9;16(Suppl 18):S9. doi: 10.1186/1471-2105-16-S18-S9 (PMC4682398; doi:10.1186/1471-2105-16-S18-S9)
Supplement: Additional file 3 — Table S2 (*.pdf). Complete list of features after two-staged feature selection using mRMR and incremental feature selection. [file 1471-2105-16-S18-S9-S3.pdf]

Table S2. Complete list of features after two-staged feature selection using mRMR and incremental feature selection.

Order	Feature	Position
1	M presence	-1
2	PSSM score of K	-7
3	I/V/L/M presence	+8
4	Termini	-
5	D/E occurrence count in a window	-
6	R presence	-3
7	I presence	-5
8	Hydrophobicity	-2
9	A presence	-7
10	V presence	-4
11	H presence	+7
12	L presence	-5
13	PSSM score of M	+10
14	R/K/H presence	+3
15	L presence	+10
16	N presence	+8
17	L presence	+1
18	D/E presence	-4
19	R presence	+2
20	Window disorder (binary)	-
21	E presence	-3
22	R/K/H presence	+4
23	Q/N presence	+1
24	S presence	-10
25	Ratio of average Y or F occurences in a window to whole protein	-
26	I presence	-2
27	G presence	+5
28	M presence	+1
29	E presence	+10
30	Q presence	+5
31	A presence	-8
32	A presence	-6
33	V presence	-7
34	W occurrence count in a window	-
35	K presence	+5
36	R/K/H presence	-3
37	S/T presence	+7
38	Q/N presence	-2
39	Q/N presence	+8
40	Ratio of average Q occurences in a window to whole protein	-
41	I occurrence count in a window	-
42	G presence	-2
43	PSSM score of D	-2
44	D presence	+1
45	G presence	-5
46	A presence	-7
47	S presence	+8
48	V presence	-3
49	R presence	-1
