# Supplementary material for: Prediction of neddylation sites from protein sequences and sequence-derived properties
Source: BMC Bioinformatics. 2015 Dec 9;16(Suppl 18):S9. doi: 10.1186/1471-2105-16-S18-S9 (PMC4682398; doi:10.1186/1471-2105-16-S18-S9)
Supplement: Additional file 4 — Figure S2 (*.pdf). Mean classification AUC using different class weights for SVM training. [file 1471-2105-16-S18-S9-S4.pdf]

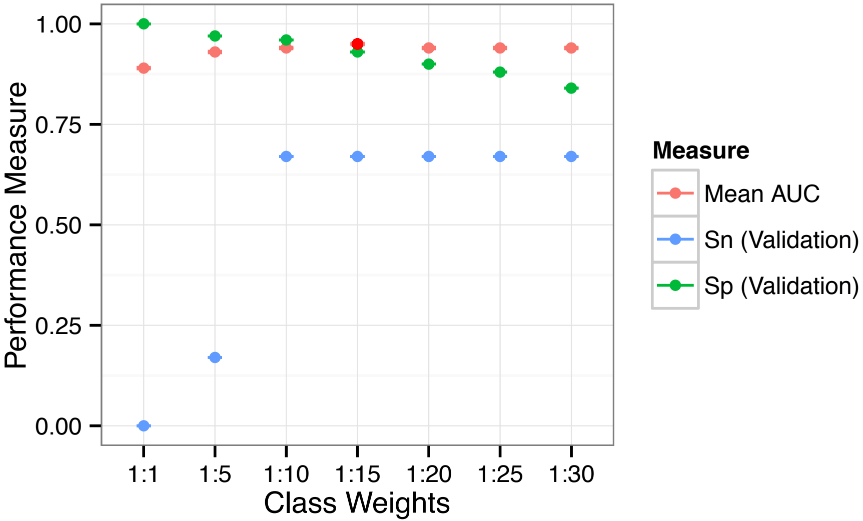

Fig. S2. Mean classification AUC using different class weights for SVM training. Under fixed window size and number of features, efficacy of using various class weights were tested to overcome the class imbalance problem. Mean classification AUC of 100 repeats of 5-fold stratified cross-validation was reported, and two standard errors are represented by error bars. Additionally, sensitivity and specificity of validation set prediction performances were reported. Highest mean accuracy with lowest standard error and highest validation sensitivity was reached at 1:15.
