# Supplementary material for: Prediction of neddylation sites from protein sequences and sequence-derived properties
Source: BMC Bioinformatics. 2015 Dec 9;16(Suppl 18):S9. doi: 10.1186/1471-2105-16-S18-S9 (PMC4682398; doi:10.1186/1471-2105-16-S18-S9)
Supplement: Additional file 5 — Table S3 (*.pdf). Summary of post-translational modification sites obtained from dbPTM. [file 1471-2105-16-S18-S9-S5.pdf]

Table S3. Summary of post-translational modification sites obtained from dbPTM.

	Ubiquitylation		Sumoylation	
**Organism**	**# of Sites**	**# of Proteins**	**# of Sites**	**# of Proteins**
A. thaliana	116	52	8	5
M. musculus	212	85	100	56
R. norvegicus	38	13	15	12
S. cerevisiae	222	125	24	14
**TOTAL**	**588**	**275**	**147**	**87**
