# Supplementary material for: Prediction of neddylation sites from protein sequences and sequence-derived properties
Source: BMC Bioinformatics. 2015 Dec 9;16(Suppl 18):S9. doi: 10.1186/1471-2105-16-S18-S9 (PMC4682398; doi:10.1186/1471-2105-16-S18-S9)
Supplement: Additional file 6 — Table S4 (*.pdf). Validation set performance under different decision thresholds. [file 1471-2105-16-S18-S9-S6.pdf]

Table S4. Validation set performance under different decision thresholds. Low, medium and high thresholds were highlighted with bold font.

Threshold	Acc	Sp	Sn	MCC
-2.00	0.35	0.31	1.00	0.15
-1.90	0.39	0.37	0.83	0.09
-1.80	0.41	0.39	0.83	0.10
-1.70	0.45	0.43	0.83	0.12
-1.60	0.47	0.45	0.83	0.12
-1.50	0.52	0.50	0.83	0.15
-1.40	0.55	0.54	0.83	0.16
-1.30	0.59	0.57	0.83	0.18
-1.20	0.60	0.59	0.83	0.19
-1.10	0.61	0.60	0.83	0.19
-1.00	0.65	0.64	0.83	0.21
**-0.90**	**0.71**	**0.70**	**0.83**	**0.25**
-0.80	0.74	0.74	0.67	0.20
-0.70	0.76	0.77	0.67	0.21
-0.60	0.78	0.78	0.67	0.23
-0.50	0.80	0.81	0.67	0.25
-0.40	0.83	0.83	0.67	0.28
-0.30	0.85	0.86	0.67	0.31
-0.20	0.86	0.87	0.67	0.32
-0.10	0.89	0.90	0.67	0.38
**0.00**	**0.90**	**0.91**	**0.67**	**0.39**
0.10	0.93	0.94	0.67	0.46
0.20	0.93	0.95	0.50	0.37
0.30	0.93	0.96	0.50	0.40
0.40	0.93	0.96	0.50	0.40
0.50	0.94	0.97	0.50	0.43
0.60	0.94	0.97	0.50	0.43
0.70	0.94	0.97	0.50	0.43
0.80	0.93	0.97	0.33	0.30
0.90	0.95	0.98	0.33	0.38
**1.00**	**0.96**	**0.99**	**0.33**	**0.45**
1.10	0.95	0.99	0.17	0.27
1.20	0.94	0.99	0.00	-0.02
1.30	0.94	0.99	0.00	-0.02
1.40	0.94	0.99	0.00	-0.02
1.50	0.94	0.99	0.00	-0.02
1.60	0.94	0.99	0.00	-0.02
1.70	0.94	0.99	0.00	-0.02
1.80	0.94	0.99	0.00	-0.02
1.90	0.94	0.99	0.00	-0.02
2.00	0.94	0.99	0.00	-0.02
