# Supplementary material for: Prediction of neddylation sites from protein sequences and sequence-derived properties
Source: BMC Bioinformatics. 2015 Dec 9;16(Suppl 18):S9. doi: 10.1186/1471-2105-16-S18-S9 (PMC4682398; doi:10.1186/1471-2105-16-S18-S9)
Supplement: Additional file 7 — Figure S3 (*.pdf). Species-specific Venn diagrams showing the number of sites modified by multiple ubiquitin-like modifications at the same site. [file 1471-2105-16-S18-S9-S7.pdf]

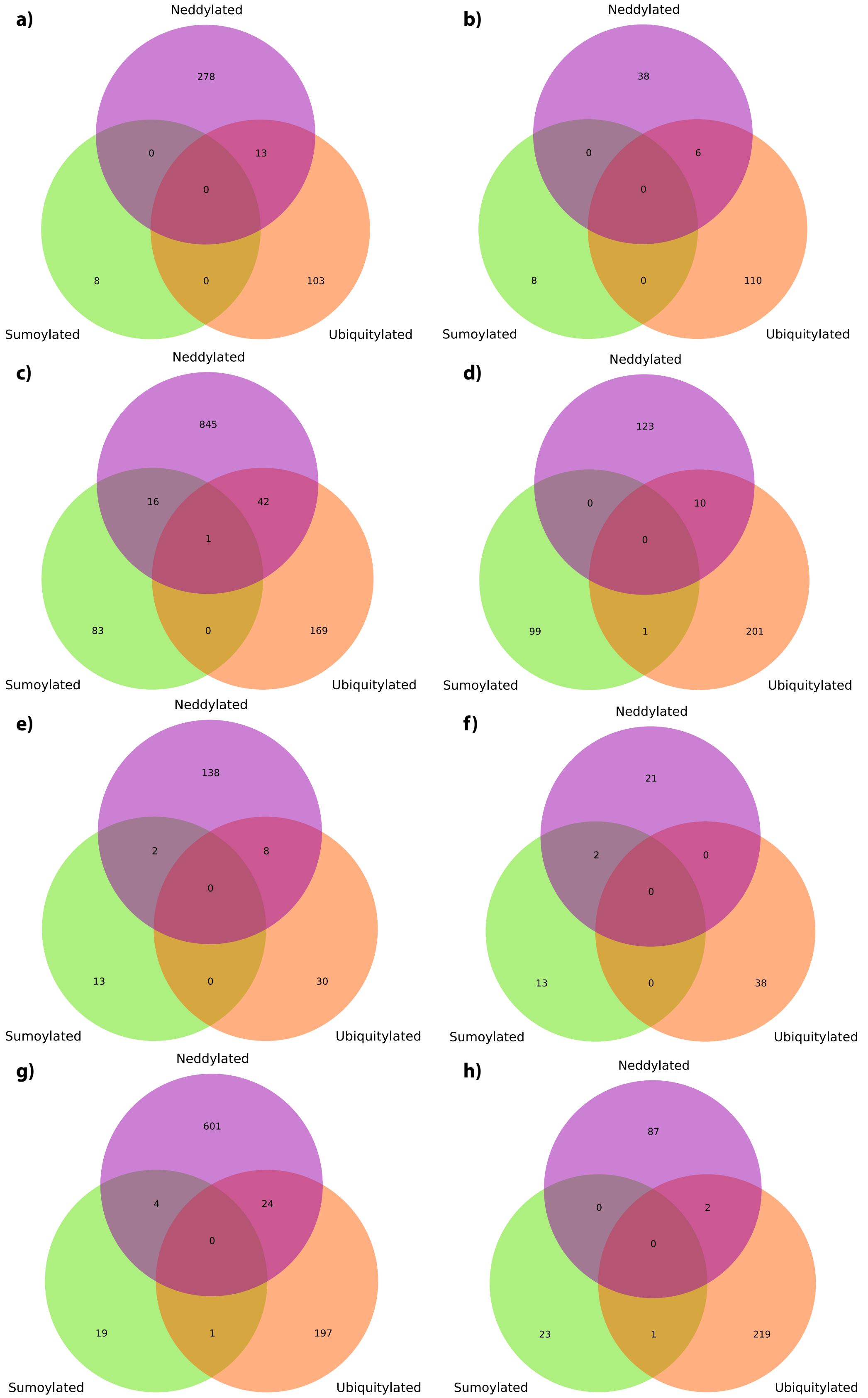

Fig. S3. Species-specific Venn diagrams showing the number of sites modified by multiple ubiquitin-like modifications at the same site. a) *A. thaliana* (neddylation prediction threshold: medium) b) *A. thaliana* (neddylation prediction threshold: high) c) *M. musculus* (neddylation prediction threshold: medium) d) *M. musculus* (neddylation prediction threshold: high) e) *R. norvegicus* (neddylation prediction threshold: medium) f) *R. norvegicus* (neddylation prediction threshold: high) g) *S. cerevisiae* (neddylation prediction threshold: medium) h) *S. cerevisiae* (neddylation prediction threshold: high)
