# Supplementary material for: Prediction of neddylation sites from protein sequences and sequence-derived properties
Source: BMC Bioinformatics. 2015 Dec 9;16(Suppl 18):S9. doi: 10.1186/1471-2105-16-S18-S9 (PMC4682398; doi:10.1186/1471-2105-16-S18-S9)
Supplement: Additional file 8 — Table S5 (*.pdf). Complete list of neddylated sites utilized in this study. [file 1471-2105-16-S18-S9-S8.pdf]

Table S5. Complete list of neddylated sites utilized in this study.

Protein	Species	Modifier	UniProt ID	Site	PMID
A4	*H. sapiens*	NEDD8	P05067-4	K676	18096514
A4	*H. sapiens*	NEDD8	P05067-4	K688	18096514
CUL1	*A. thaliana*	RUB1	Q94AH6	K682	10611386, 15208391
CUL1	*H. sapiens*	NEDD8	Q13616	K720	10713156, 10772955, 10921923, 21145461, 24949976
CUL1	*H. sapiens*	NEDD8	Q13616	K769	18247557
CUL1	*H. sapiens*	NEDD8	Q13616	K472	21249194
CUL2	*H. sapiens*	NEDD8	Q13617	K689	10092517, 15966899, 17254749, 23401859
CUL2	*H. sapiens*	NEDD8	Q13617	K719	22537386
CUL3A	*A. thaliana*	RUB1	Q9ZVH4	K679	15618422
CUL4	*S. pombe*	RUB1	O14122	K680	10880460
CUL5	*H. sapiens*	NEDD8	Q93034	K724	20682250
CUL3	*D. melanogaster*	NEDD8	Q9V475	K717	15843622, 16127432
E2F1	*H. sapiens*	NEDD8	Q01094	K117	22836579
E2F1	*H. sapiens*	NEDD8	Q01094	K120	22836579
E2F1	*H. sapiens*	NEDD8	Q01094	K125	22836579
E2F1	*H. sapiens*	NEDD8	Q01094	K185	22836579
ELAV1	*H. sapiens*	NEDD8	Q15717	K283	22095636
ELAV1	*H. sapiens*	NEDD8	Q15717	K313	22095636
ELAV1	*H. sapiens*	NEDD8	Q15717	K326	22095636
H4	*H. sapiens*	NEDD8	P62805	K6	23394999
H4	*H. sapiens*	NEDD8	P62805	K9	23394999
H4	*H. sapiens*	NEDD8	P62805	K13	23394999
H4	*H. sapiens*	NEDD8	P62805	K17	23394999
H4	*H. sapiens*	NEDD8	P62805	K21	23394999
H4	*H. sapiens*	NEDD8	P62805	K32	23394999
P53	*H. sapiens*	NEDD8	P04637	K370	15242646
P53	*H. sapiens*	NEDD8	P04637	K372	15242646
P53	*H. sapiens*	NEDD8	P04637	K373	15242646
P53	*H. sapiens*	NEDD8	P04637	K320	17098746
P53	*H. sapiens*	NEDD8	P04637	K321	17098746
P73	*H. sapiens*	NEDD8	O15350	K321	16980297
P73	*H. sapiens*	NEDD8	O15350	K327	16980297
P73	*H. sapiens*	NEDD8	O15350	K331	16980297
RCAN1	*H. sapiens*	NEDD8	P53805	K96	23118980
RCAN1	*H. sapiens*	NEDD8	P53805	K104	23118980
RCAN1	*H. sapiens*	NEDD8	P53805	K107	23118980
VHL	*H. sapiens*	NEDD8	P40337	K159	15060148
VHL	*H. sapiens*	NEDD8	P40337	K171	15060148
VHL	*H. sapiens*	NEDD8	P40337	K196	15060148

DLG4	*M. musculus*	NEDD8	Q62108	K202	25581363
SHC1	*M. musculus*	NEDD8	P98083-2	K3	23267066
CDC53	*S. cerevisiae*	RUB1	Q12018	K760	11027288, 18206966, 20832729
CUL8	*S. cerevisiae*	RUB1	P47050	K791	14519104
CUL3	*S. cerevisiae*	RUB1	P53202	K688	14519104
CUL3	*S. pombe*	RUB1	Q09760	K729	11504566
ANIA_10008	*E. nidulans*	NEDD8	C8VRE7	K826	21119001
TGFR2	*H. sapiens*	NEDD8	P37173	K556	23290524
TGFR2	*H. sapiens*	NEDD8	P37173	K567	23290524
BRAP	*H. sapiens*	NEDD8	Q7Z569	K432	23554956
H2A1B	*H. sapiens*	NEDD8	P04908	K119	23554956
H2A1B	*H. sapiens*	NEDD8	P04908	K120	24634510
