# Supplementary material for: Prediction of neddylation sites from protein sequences and sequence-derived properties
Source: BMC Bioinformatics. 2015 Dec 9;16(Suppl 18):S9. doi: 10.1186/1471-2105-16-S18-S9 (PMC4682398; doi:10.1186/1471-2105-16-S18-S9)
Supplement: Additional file 9 — Table S6 (*.pdf). Sezerman grouping of amino acids. [file 1471-2105-16-S18-S9-S9.pdf]

Table S6. Sezerman grouping of amino acids.

**Groups**
IVLM
RKH
DE
QN
ST
A
G
W
C
YF
P
