# Supplementary material for: Prediction of neddylation sites from protein sequences and sequence-derived properties
Source: BMC Bioinformatics. 2015 Dec 9;16(Suppl 18):S9. doi: 10.1186/1471-2105-16-S18-S9 (PMC4682398; doi:10.1186/1471-2105-16-S18-S9)
Supplement: Additional file 10 — Supplementary Information (*.pdf). Supplementary methods can be found in this file. [file 1471-2105-16-S18-S9-S10.pdf]

Supplementary Information

Prediction of neddylation sites from protein sequences and sequence-derived properties

Ahmet Sinan Yavuz[1], Namık Berk Sözer[2] and Osman Uğur Sezerman[3,*]

[1]Biological Sciences and Bioengineering Program, Faculty of Engineering and Natural Sciences, Sabancı University, Tuzla, Istanbul, 34956, Turkey.

[2]Department of Genetics and Bioengineering, Faculty of Engineering and Architecture, Yeditepe University, Ataşehir, Istanbul, 34755, Turkey.

[2]Department of Biostatistics and Medical Informatics, Faculty of Medicine, Acıbadem University, Ataşehir, Istanbul, 34752, Turkey.

* Corresponding author: Osman Uğur Sezerman (ugur.sezerman@acibadem.edu.tr)

Performance Evaluation

In order to assess the performance of the classification model, we have calculated various statistics. For each evaluation strategy, Matthew's correlation coefficient (MCC), sensitivity (Sn), specificity (Sp) and accuracy (Ac) are reported with equations (1-4).

$$MCC = \frac{(TP*TN)-(FN*FP)}{\sqrt{(TP+FN)*(TN+FP)*(TP+FP)*(TN+FN)}} \quad (1)$$

$$Sn = \frac{TP}{TP+FN} \quad (2)$$

$$Sp = \frac{TN}{TN+FP} \quad (3)$$

$$Ac = \frac{TP+TN}{TP+FP+TN+FN} \quad (4)$$

where TP represents the number of correctly predicted neddylated windows; TN indicates correctly predicted non-neddylated windows; FP denotes over-predicted windows, and FN represents under-predicted windows.

In order to prevent overlearning in performance evaluation, we have used stratified cross-validation approach. Cross-validation is a statistical evaluation technique for measuring the performance of a predictive model. In N-fold cross-validation, the entire set is randomly divided into N subsets. A single subset of these N subsets is retained as the test set and the remaining N-1 subsets are used as training sets. This cross-validation process is repeated for N times with each subset used exactly once as a testing set. In stratified cross-validation, the percentage of samples was aimed to be preserved for each class at each fold. As a main evaluation strategy, 5-fold stratified cross-validation was selected. Secondary evaluation strategy was selected as validation set.
